# Influence of External Gaseous Environments on the Electrical Properties of ZnO Nanostructures Obtained by a Hydrothermal Method

**DOI:** 10.3390/nano6120227

**Published:** 2016-11-29

**Authors:** Marcin Procek, Tadeusz Pustelny, Agnieszka Stolarczyk

**Affiliations:** 1 Department of Optoelectronics, Silesian University of Technology, 2 Krzywoustego St., 44-100 Gliwice, Poland; tadeusz.pustelny@polsl.pl; 2 Department of Physical Chemistry and Technology of Polymers, Silesian University of Technology, 9 Strzody St., 44-100 Gliwice, Poland; agnieszka.stolarczyk@polsl.pl

**Keywords:** zinc oxide nanostructures, electrical gas sensors, electrical resistivity of ZnO nanostructures, nitrogen dioxide detection, ultraviolet excitation of semiconductors

## Abstract

This paper deals with experimental investigations of ZnO nanostructures, consisting of a mixture of nanoparticles and nanowires, obtained by the chemical (hydrothermal) method. The influences of both oxidizing (NO_2_) and reducing gases (H_2_, NH_3_), as well as relative humidity (RH) on the physical and chemical properties of ZnO nanostructures were tested. Carrier gas effect on the structure interaction with gases was also tested; experiments were conducted in air and nitrogen (N_2_) atmospheres. The effect of investigated gases on the resistance of the ZnO nanostructures was tested over a wide range of concentrations at room temperature (RT) and at 200 °C. The impact of near- ultraviolet (UV) excitation (λ = 390 nm) at RT was also studied. These investigations indicated a high response of ZnO nanostructures to small concentrations of NO_2_. The structure responses to 1 ppm of NO_2_ amounted to about: 600% in N_2_/230% in air at 200 °C (in dark conditions) and 430% in N_2_/340% in air at RT (with UV excitation). The response of the structure to the effect of NO_2_ at 200 °C is more than 10^5^ times greater than the response to NH_3_, and more than 10^6^ times greater than that to H_2_ in the relation of 1 ppm. Thus the selectivity of the structure for NO_2_ is very good. What is more, the selectivity to NO_2_ at RT with UV excitation increases in comparison at elevated temperature. This paper presents a great potential for practical applications of ZnO nanostructures (including nanoparticles) in resistive NO_2_ sensors.

## 1. Introduction

Within the group of nanostructured semiconductors with a wide band gap, zinc oxide (ZnO) is a material which has been comprehensively investigated due to its interesting physical and chemical properties and the possibility of its potential extensive application in solar cells, ultraviolet (UV) photodetectors, and light emitters [1,2,3,4]. ZnO is an n-type semiconductor with a band-gap exceeding 3 eV. ZnO crystallizes in three forms: hexagonal (wurtzite), regular (zinc blende), and rarely observed regular (rock-salt) [5]. The wurtzite structure [6,7] is the most stable and the most common form of ZnO and it is most often used in ZnO based gas sensors [8]. The wurtzite structure belongs to the spatial group of C^4^_6V_. The tetrahedral system of nodes is typical for covalent binding with sp^3^ hybridization. In the case of ZnO, the binding type has only partly covalent character, with participation of ion bindings. [9]. The conduction band of ZnO is mainly formed by orbitals 4s Zn^2+^, and the valence band forms 2p O^2−^. ZnO, like other semiconductor compounds from the II–VI group, has a simple energetic band-gap.

There are many approaches of synthesis of ZnO nanostructues described in the literature. Primary synthesis methods include chemical vapor deposition (CVD) [10], metal organic CVD [11], thermal evaporation [12], the template method [13], electrochemical deposition [14], and the laser ablation method [15]. In recent years much interest has been devoted to ZnO nanostructures obtained by means of chemical methods, mainly participation, sol-gel, and hydrothermal methods [16,17,18,19,20,21,22,23,24,25].

Detection and measurement of concentrations of toxic gases such as NO_2_ or NH_3_ and explosive gases e.g. H_2_ have become a necessity due to environmental protection and public safety [26,27]. Also the humidity effect on the gas sensing structures has to be taken into account due to its different values under real operating conditions [28]. Many publications emphasized the potential of ZnO nanostructures in gas sensors [8,25,27,29,30].

ZnO nanostructures gas sensing properties have been tested both theoretically and experimentally [31,32,33,34,35]. The investigations concerning gas sensors based on ZnO are already well advanced [20,29,36]. Generally, those structures are tested at elevated temperature (from 200 °C even up to 700 °C) [18,36].

Due to the extended surface of the ZnO nanostructures it ought to be stressed that their electrical properties depend considerably on the surface phenomena and electrical contact between them. On the surface of ZnO nanostructures, energy states result from the breaking of chemical bonds and creation of oxygen vacancies. The surface potential (*V_s_*) of the nanostructure determines the curvature of the energy bands on its surface. It follows that between nanostructures the potential barriers, which determine the character of the flow of an electric current, are formed. The classical division of nanostructures distinguishes big grains, where *z_g_* > λ_D_ and small grains *z_g_* < λ_D_, where *z_g_* indicates the size of nanograins and λ_D_ is the Debye’s shielding radius [31]. The shielding radius λ_D_ depends strongly on the concentration of electrons in the bulk *n_B_*. In the case of ZnO nanoparticles proceeds: λ_D_ = 1 nm for *n_B_* = 10^18^ cm^−3^; λ_D_ = 3 nm for *n_B_* = 10^17^ cm^−3^; λ_D_ = 10 nm for *n_B_* = 10^16^ cm^−3^.

The conductivity in structures comprising both large and small grains is a combination of so-called over-barrier and channel conductivities [34]. Theoretical analyses [34,37] indicate that the electric conductivity of a semiconductor nanostructure decreases exponentially with increasing surface potential barriers (e*V_s_)*.

The present paper deals with ZnO nanostructures obtained by means of the hydrothermal method composed of nanograins and nanowires (small and large grains). The aim of our study was to investigate the electrical properties of such nanostructures in different gaseous and external conditions. The resistance of ZnO nanostructures affected by various gaseous environments (oxidizing NO_2_ and reducing gases: H_2_, NH_3_) was tested. The effects of carrier gas (nitrogen and air), humidity, temperature, and electromagnetic irradiation to the resistance of the structure were examined. The investigated material showed promising NO_2_ sensing properties at RT with ultraviolet excitation and at elevated temperature (200 °C).

With this approach, objective results and conclusions were obtained about the influence of gaseous environments, not only qualitatively, but also quantitatively. Thus, the results of the experimental investigations are of particular importance.

## 2. Experimental

### 2.1. Synthesis of ZnO Nanostructures

ZnO nanostructures were obtained by means of the hydrothermal method with ethylenediamine precursor (EDA). The following materials were used: Zn(NO_3_)_2_·6H_2_O, EDA (produced by Simga Aldrich, Saint Louis, MO, USA). NaOH, and ethanol (produced by POCH, Gliwice, Poland).

In a teflon vessel, 0.600 g NaOH and 0.440 g Zn(NO_3_)_2_·6H_2_O were dissolved in 3 mL of deionized water. Then 30 mL ethanol, 5 mL deionized water, and 5 mL EDA were added. Under these conditions ZnEDA complex was formed according to the reaction (Equation (1)) [38].

Zn^2+^ + 3H_2_NCH_2_CH_2_NH_2_ ↔ [Zn(H_2_NCH_2_CH_2_NH_2_)_3_]^2+^ (1)

The solution was placed for 30 min in an ultrasonic bath and then passed to an autoclave. The reaction was run for two hours at a temperature of 180 °C. Above 100 °C decomposition of ZnEDA complex occurs due to chemical equilibrium shift to the left side of the reaction (Equation (1)) [38]. Elevated temperature causes the hydrolysis of EDA and increases OH^−^ concentration (Equation (2)).

H_2_NCH_2_CH_2_NH_2_ + 2H_2_O → H_3_NCH_2_CH_2_NH_2_ + 2OH^−^ (2)

In such a hydrothermal process Zn(OH)_2_ and next ZnO were formed according to (Equation (3)).

Zn^2+^ + OH^−^ → Zn(OH)_2_ → ZnO (3)

After the reaction in the autoclave, the product was cooled down, the sediment was centrifuged, repeatedly rinsed in deionized water and ethanol. The zinc oxide was dried for 12 h under reduced pressure (15 mbar) at a RT.

### 2.2. Material Characterization Details

After the syntheses, the ZnO structures were analyzed by means of a field emission scanning electron microscope (FE-SEM) SUPRA 35 (Carl ZEISS, Jena, Germany).

To explain the molecular nature of the resulting ZnO nanostructures, they were tested by applying Raman spectroscopy. Raman investigations were made using the Ntegra Spectra system (NT-MDT, Moscow, Russia), a microscope equipped with a CCD (Charge Coupled Device) detector using green (532 nm) laser excitation.

To determine the crystal nature of the ZnO nanostructures, X-ray powder diffraction (XRD) measurements were made. XRD was investigated using a diffractometer X’Pert Pro MPD (PANAlytical, Almelo, Netherlands), containing a cobalt lamp with the main wavelength of 0.179 nm (K-alpha line) in its spectrum.

For the inspection of the grains’ distribution on the transducer a scanning electron microscope (SEM) Inspect S50 (FEI, Hillsboro, OR, USA) was used.

### 2.3. Fabrication of Resistive Structures Based on ZnO Nanostructures

In order to investigate the electrical properties of ZnO nanostructures, a resistive structure on an interdigital transducer (IDT) was made. ZnO nanostructures were deposited on an IDT by means of a drop coating method. The transducer was obtained using the photolithography method, from a 300 nm thick gold layer on a silicon substrate with a 1 μm SiO_2_ layer. The width of the electrodes (including the distance from each other) was 5 μm. The dimensions of the transducer were 10 × 10 mm. For the purpose of depositing ZnO nanostructures on the substrate, they were dispersed in pure hexane (produced by POCH, Gliwice, Poland) by means of an ultrasonic bath (the mixing lasted 5 min). Next the suspension was dropped onto the transducer and dried at RT. The material not adhering to the substrate was removed using compressed air with a pressure of 10 bar. Prior to the measurements, the structure was heated up to a temperature of 200 °C to remove the retained dispergent and to let the structure dry.

Three such sensing structures were fabricated and tested, all of them gave similar results and had similar properties. In order to compare the reaction of the ZnO nanostructures affected by external atmospheres, temperature and light excitation the results for the same structure are presented in the text.

### 2.4. Gas Measurement Details

**Figure 1:**
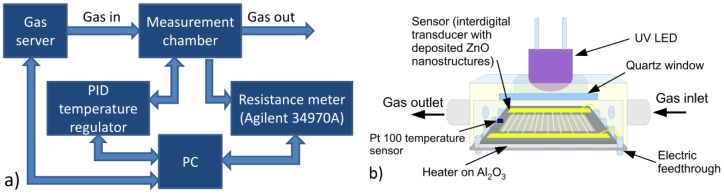
Scheme of (**a**) the measurement stand and (**b**) the measurement chamber.

ZnO nanostructures were investigated in various gaseous atmospheres and under various environmental conditions (at different temperature and UV lighting). This required a special measuring chamber, in which the transducer with the deposited ZnO nanostructures was placed (Figure 1b). This measuring chamber can heat the structure by means of a thick-layer heater on an Al_2_O_3_ substrate and can measure the temperature by a Pt-100 sensor. A quartz window and an ultraviolet LED (λ ≈ 390 nm) permits the illumination of the sample by means of UV radiation. The transducer mounted on the heater was mobilized on a special chip, and the contacts between its leg and the transducer electrodes were constructed by means of wire bonding (gold wire with a diameter of 25 μm) using the bonder 53XX-BDA (made by F&K DELVOTEC, Braunau, Austria). The cover of this chamber consists of PTFE and is equipped with fast gas connectors of stainless steel. The block scheme of the measurement setup is shown in Figure 1a. The acquisition of data and control of the measurements were accomplished by a PC. The temperature was controlled and measured by the PID controller SR94 (produced by Shimaden, Tokyo, Japan). In all these cases, the resistance was measured making use of a multichannel switch unit 34970A (produced by AGILENT, Santa Clara, CA, USA).

The applied gaseous mixtures were produced by a gas server allowing precise preparation of the gaseous atmospheres, composed of any kind of carrier gas and up to five different gases of precisely determined concentrations. The gas server was described in an earlier paper [39].

The flow of gas through the measuring chamber was constant for all the measurements (500 mL/min). During the measurements two different gases were applied as the atmosphere (carrier gases), viz. nitrogen 6.0 and synthetic air (O_2_: 20%; N_2_: 80%). Their relative humidity was the same, amounting to about: 6% ± 1% (at 22 °C). In the experiments, adequate standard gases were used, viz. H_2_: 99.9994%; NO_2_: 100 ppm in N_2_; NH_3_: 500 ppm in air. The humidity of the gas was changed by means of a bubble humidifier and monitored by an RH meter. The measurement of the response of the ZnO nanostructures to the effect of the gases consisted in alternating the introduction of pure carrier gas into the measuring chamber and the tested gas with the given concentration in the atmosphere (synthetic air or nitrogen N_2_). In each successive cycle, the concentration of the gas was raised. The measurements were performed at RT = 23 °C, at elevated temperature of *T* = 200 °C and also when illuminating the sample of ZnO nanostructure by UV light at RT.

The structure responses to a given gases were calculated according to the formula:

(4) $$Response=\frac{R_{g}-R_{a}}{R_{a}}\cdot 100\%,$$

where *R_a_* is the sensor resistance in the carrier gas and *R_g_* is the sensor resistance after the action with the selected gas.

### 2.5. Details Concerning Spectral Measurements

The dependence of the resistance of ZnO nanostructures on the wavelength of optical excitation was measured. The resistance of the ZnO nanostructures was measured during illumination with light in the range from 300 nm to 750 nm. The source of light was a xenon lamp, and the wavelength was measured by tuning the monochromator DH-10 (made by Jobin Yvon, Lonjumeau, France). In this case, the measurements were performed in atmospheric air, at RT. The intensity of light was kept at a constant level and monitored by a spectrometer HR 2000 + ES (made by Ocean Optics, Dunedin, FL, USA).

### 2.6. Details Concerning the Temperature Measurements

Investigations of the dependence of the resistance of ZnO nanostructures on temperature *R*(*T*) in various gaseous atmospheres, viz. synthetic air and nitrogen, were performed. In each series of tests, the sample was prepared by heating in a given carrier gas up to 300 °C and then cooling down to RT. The resistance was recorded for other temperature cycles of heating and cooling in the given atmosphere. The temperature was changed within the range of 40–300 °C every 10 °C until the resistance became stable (the interval of time was equal to about *Δt* = 2 min).

## 3. Results and Discussion

### 3.1. ZnO Structures Characterization

**Figure 2:**
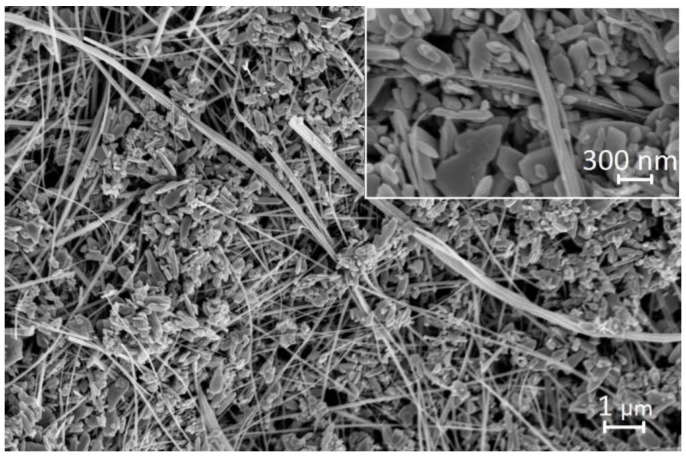
Field emission scanning electron microscope (FE-SEM) image of the ZnO nanostructures (magnification 20,000× and 50,000×).

Morphology of the ZnO structures is presented on FE-SEM images in Figure 2. The nanostructures of ZnO obtained by the hydrothermal method constitute a rich mixture of different geometrical forms, from nanobars and nanotubes, most with lengths up to a few µm up to nanoparticles such as nanolumps with sizes of a few nm. From the physical point of view, a division of nanostructures into the so-called large and small ones is reasonable [31]. In our case, the large geometrical variety of ZnO nanostructures and the wide range of their sizes require them to be treated as a mixture of both large and small nanostructures.

**Figure 3:**
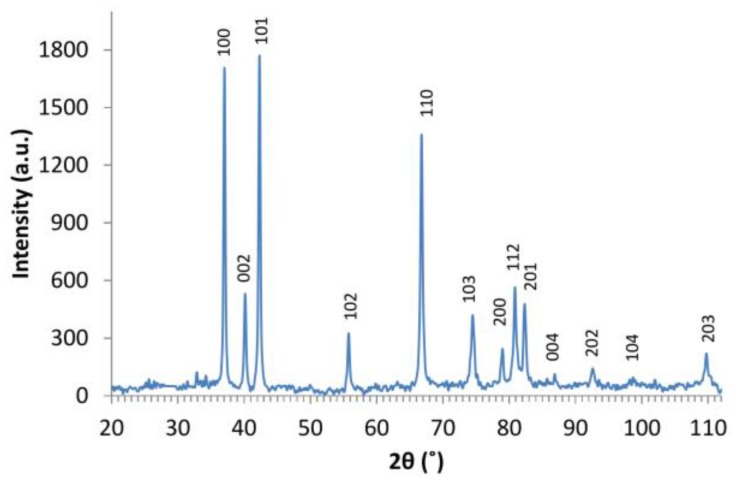
X-ray powder diffraction (XRD) patterns of ZnO nanostructures (λ = 0.179 nm).

The XRD results are shown in Figure 3. The obtained diffractogram is characteristic for the hexagonal structure of ZnO with the spatial group P63mc (patterns are consistent with the ICSD card at the code No. 180052 titled ”Zinc Oxide–Nanoparticles”). In the spectrum, no additional peaks could be detected. The average crystal sizes estimated from XRD patterns using the Scherer equation [40] are in the range of 227 nm (for crystallographic plane (100)) to 3 nm (for plane (203)). The obtained results confirm conclusions from FE-SEM where mixtures of structures with different shapes were observed.

**Figure 4:**
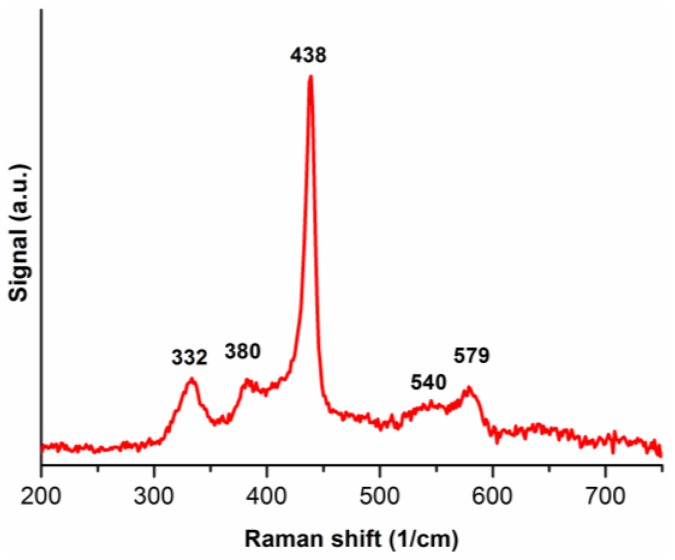
Raman spectrum of ZnO nanostructures.

Figure 4 presents the Raman spectrum of ZnO nanostructures. The obtained spectrogram is compatible with Raman peak values for ZnO nanocrystals given in the reference [6] with slight drift (in the range of 2–5 cm^−1^) caused by different crystals sizes. In this case all the atoms occupy C_3v_ sites. Group theory shows that active Raman modes are A1+E1+2E2. The phonons of A1 and E1 symmetry are polar phonons and, hence, exhibit different frequencies for the transverse-optical (TO) and longitudinal-optical (LO) phonons [6]. Raman modes at 332, 380, 438, 540 and 579 cm^−1^ are denoted as 2E2, E1(TO), E2, A1(LO) and E1(LO) modes, respectively.

**Figure 5:**
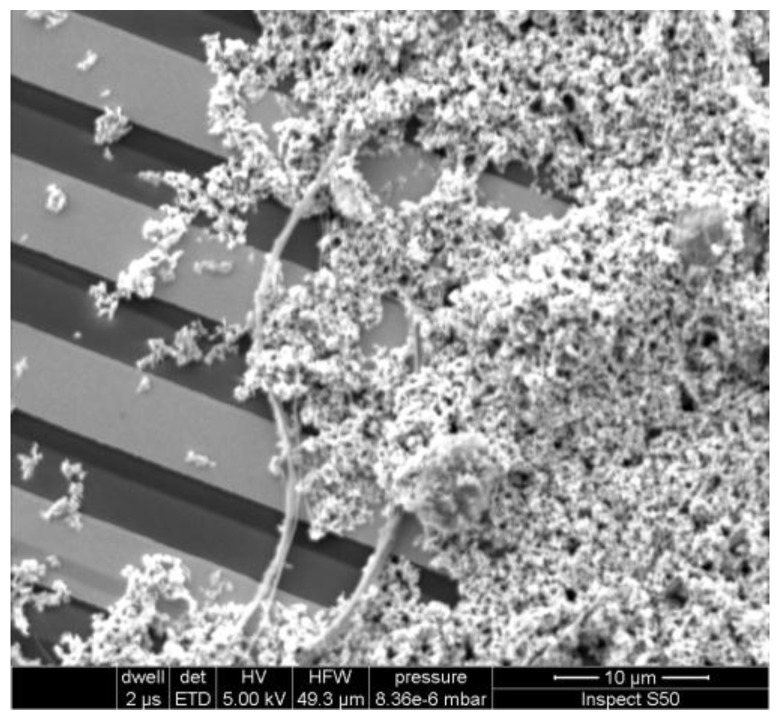
Scanning electron microscope (SEM) image of the distribution of ZnO nanostructures on the interdigital transducer.

ZnO nanostructures deposited on the IDT have a relatively uniform distribution and good adhesion to the substrate, as may be observed on the SEM image in Figure 5. The adhesion of the nanostructures to the transducer is the result of van der Waals and electrostatic forces. Thus, small grains which have smaller mass to contact area ratio have better adhesion than bigger ones. Long nanowires which have contact to a few electrodes stabilized the electrical properties of the sensor structure. Thanks to the mixture of small grains and nanowires, layers with good adhesion and electrical stability were obtained. The thickness of the layer is not uniform and its value is in the range from single crystalline size up to single micrometers (Figure 5).

### 3.2. Investigations of the Influence of Light on the Resistance of ZnO Nanostructures

**Figure 6:**
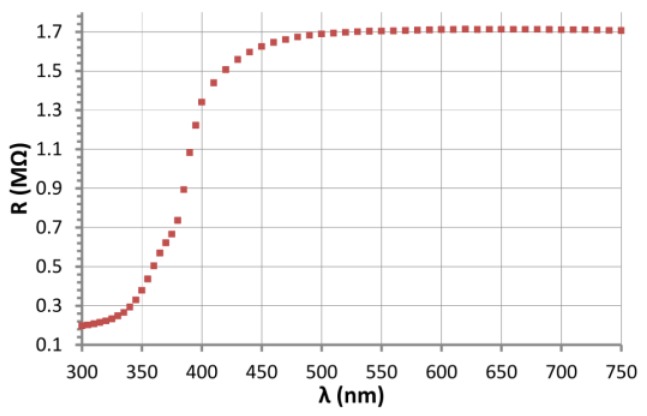
The dependence of the resistance of ZnO nanostructures on the wavelength.

The results of spectral tests *R*(λ) are gathered in Figure 6. Changes of the resistance are observed in the range between 300 and 500 nm. Below 380 nm the resistance changes are smaller. The characteristics show the existence of an absorption edge in the ZnO nanostructures somewhere about 380 nm. In crystalline ZnO the absorption edge (corresponding to the band gap) is also observed at about 380 nm [9], which corresponds to an energy *ΔE* of 3.25 eV.

The decrease of the resistance under UV irradiation is mainly caused by the increase in the concentration of the electrical charges (electrons and holes), as a result of photogeneration as well as excitation of electrons from the surface states. Of no less importance is the phenomenon of photocatalysis leading to the desorption of the gases (especially oxygen) absorbed (in the form of molecules, atoms, and ions) on the surface active centers of the ZnO nanostructures [41].

### 3.3. The Effect of the Carrier Gas and Temperature on the Electrical Properties of ZnO Nanostructures

**Figure 7:**
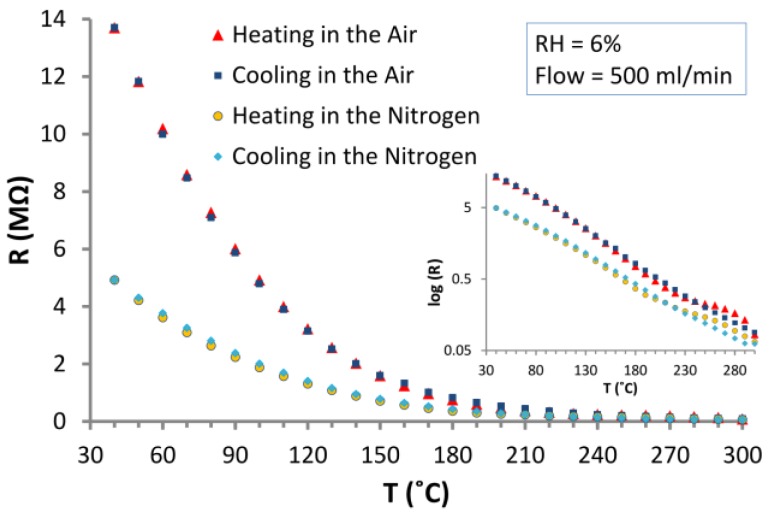
Dependence of the resistance of the structure based on ZnO nanoparticles on temperature in: synthetic air and in nitrogen (gas flow = 500 mL/min., *RH* = 6%).

The authors attempted to explain experimentally to what extent the oxygen contained in the air affects the interaction of the tested gases with ZnO nanostructures. Figure 7 illustrates the dependence of the electrical resistance on the temperature of the layer of ZnO nanostructures in atmospheres of synthetic air and in nitrogen.

The diagram of *R*(*T*) (Figure 7) shows the differences between the resistance values in both carrier gases during cooling and heating the sample. It is particularly noticeable in the temperature range from 40–180 °C. It can be seen in Figure 7 that at the measured temperature range the resistance of ZnO nanostructures is higher in air (in the presence of oxygen) than that in nitrogen. An increase in the temperature reduces the resistance of the nanostructures. It is the effect of a decrease of the concentration of electrons in the surface states and the reduction of the surface potential barriers of ZnO nanostructures. In the nitrogen atmosphere the created oxygen vacancies stay unoccupied.

### 3.4. The Reaction of ZnO Nanostructures to NO_2_

As an example of oxidizing gas small concentrations of NO_2_ (1–20 ppm) were chosen.

**Figure 8:**
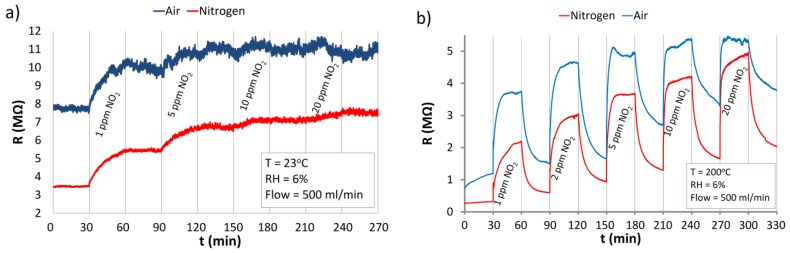
The reaction of ZnO nanostructures to NO_2_ in the atmospheres of synthetic air and nitrogen at: (**a**) RT; (**b**) elevated temperature of 200 °C.

The characteristics shown in Figure 8a prove that at RT ZnO nanostructures reacted to NO_2_ only in the first cycle (1 ppm) where their resistance increased by c.a. 50% in N_2_ and 30% in air. When the resistive ZnO structure was “saturated” its further exposure to NO_2_ action at RT did not result in a change of the resistance. At RT, when NO_2_ was removed from the carrier gas, the structures did not recover, and the resistance of the structure did not return to its initial values. The results at 200 °C are presented in Figure 8b. Heating the structure up to 200 °C resulted in a strong increase of the response of ZnO nanostructures to NO_2_. In this case responses to 1 ppm of NO_2_ were c.a. 600% in nitrogen and c.a. 230% in air. At the elevated temperature, sensor recovery is observed (Figure 8b), which is caused by the desorption of NO_2_ from the ZnO nanostructures. It is revealed by the decrease in the resistance after each NO_2_ exposure.

The elevated temperature (200 °C) of the structure leads to an increase in free chemical bonds (oxygen vacancies) on the surface of ZnO nanostructures, which can bind with gas molecules. The changes of the resistance of the structures depend also on the concentration of electrons, which are thermally transferred from traps localized in the band-gap to the conduction band. Therefore, the spatial distribution of electrical charges in the ZnO nanostructures is also changed. The temperature of about 200 °C also facilitates desorption of the NO_2_ from the active centers. The investigations showed that detoxication after 1 ppm of NO_2_ exposure of the structure requires a longer time in an atmosphere of synthetic air (*t*_90%_ = 880 s) than in nitrogen (*t*_90%_ = 700 s). In an atmosphere of air, active centers can join to both NO_2_ and oxygen simultaneously [34,42,43].

The differences between the structure reaction to NO_2_ at RT and elevated temperature can be explained by a chemical mechanism of this interactions. At RT, NO_2_ is absorbed by ZnO nanostructures mainly in the form of NO_3_^−^ ions with evacuation of gaseous NO [8,44]. At the elevated temperature, the absorbed NO_3_^−^ undergoes decomposition to gaseous NO_2_ and adsorbed O^−^. As NO_2_ and its derivatives are oxidizing gases, they incorporate electrons from the ZnO nanostructures, which increase their resistance.

**Figure 9:**
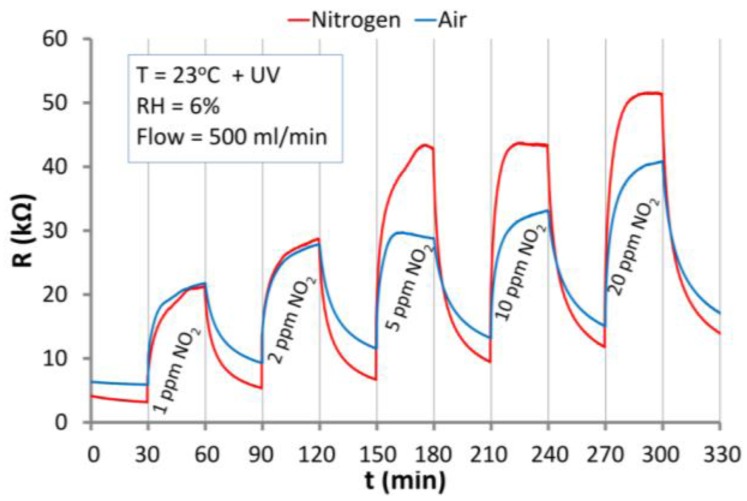
The response of the ZnO nanostructures to the effect of NO_2_ and ultraviolet (UV) irradiation in atmospheres of air and nitrogen at RT.

The results of the response of ZnO nanostructures to NO_2_ in air and nitrogen atmospheres under UV illumination and at RT are shown in Figure 9. Resistance of the structure increases proportionally to NO_2_ concentration. The structure responses to 1 ppm of NO_2_ amount to 430% in N_2_ and 340% in the air. Under this conditions in both carrier gases structure recovery occurs, which proves that UV illumination allows a desorption of NO_2_ from the ZnO nanostructures at RT.

The excitation of ZnO nanostructures by means of UV radiation causes both photogeneration of electron-hole pairs, and generation of charge carriers from the surface states. On the ZnO surface the photocatalytic reactions of the adsorption (with capture of electron) and desorption (with capture of hole) of oxygen can occur [45].

In the case of air conditions both reactions occur and the surface potential is the result of equilibrium between them. In nitrogen only oxygen desorption occurs, and thus the surface potential is smaller than in air. This can be observed in the differences of base resistances under both conditions (Figure 9). This phenomenon has also impact on the kinetics of the reaction of the structure with NO_2_. Due to the higher oxygen vacancy concentration in nitrogen the structure response is higher than under air conditions.

The presented results prove that UV excitation enables ZnO nanostructures to respond and recover at RT. Under air conditions the structure response is higher at RT with UV activation than at elevated temperature. This makes UV excited ZnO nanostructures promising materials for low concentration NO_2_ sensors operating at RT.

### 3.5. Reactions of ZnO Nanostructures with Reducing Gases

#### 3.5.1. The Reaction of ZnO Nanstructures with Hydrogen

**Figure 10:**
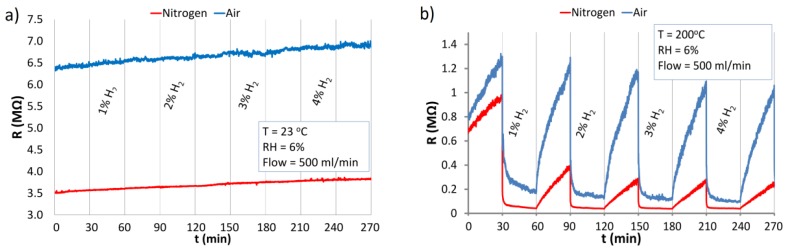
Reaction of the ZnO nanostructures with H_2_ in the atmosphere of synthetic air and nitrogen at: (**a**) RT; (**b**) temperature of 200 °C.

The reaction of ZnO nanostructures with H_2_ in an atmosphere of synthetic air and nitrogen at RT and at 200 °C is presented in Figure 10. The structure was exposed to relatively high concentrations of hydrogen in the range of a few percent (1% ≡ 10^4^ ppm). The detection of such high H_2_ concentrations is important because hydrogen is explosive at 4% concentration in air.

At RT (Figure 10a) no reaction of the ZnO nanostructures to H_2_ was observed. Contrary to RT, at elevated temperature (200 °C) under both atmospheres (Figure 10b) a relatively high and fast response to hydrogen was observed. The response is characteristic of reducing gases, which decrease the resistance of n-type semiconductors. In comparison to adsorption, the rate of desorption at 200 °C is rather slow, especially under nitrogen atmosphere. Due to the relatively high concentrations of H_2_, there are very small differences between the responses to various concentrations. A temperature of 200 °C seems to be high enough for reversible adsorption of dissociatively chemisorbed hydrogen to occur. Adsorption of H_2_ results in the formation of a H–O–ZnO surface [46]. The response of ZnO nanostructure at 200 °C is relatively fast because of the high concentration of H_2_ and the small size of hydrogen molecules. The hydrogen ions react with oxygen ions on the ZnO surfaces and may cause desorption of oxygen from active centers and the ZnO lattice [25,29]. This results in a decrease in the potential barriers between nanograins and thus the resistance of the sensor structure is reduced. The desorption process is faster in air than in nitrogen (Figure 10b). The oxygen from the air leads to a re-oxidation of the oxide vacancies on the surface of ZnO nanostructures during recovery of the structure. In the nitrogen atmosphere, the resistive structure recovers very weakly due to the absence of oxygen (or other oxidizing analytes) which can fill the vacancies created by hydrogen. Therefore the role of oxygen from the air is important in the sensor recovery process.

**Figure 11:**
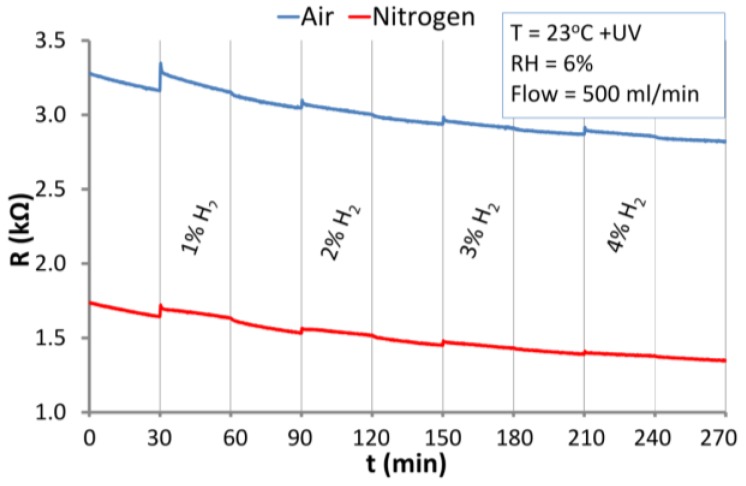
Response of the ZnO nanostructures to H_2_ and at UV irradiation under atmospheres of air and nitrogen at RT.

The reaction of ZnO nanostructures to H_2_ under continuous UV irradiation at RT is presented in Figure 11. Similar to dark conditions (Figure 10a), the resistive structure practically does not react to hydrogen at RT. Under UV radiation in both carrier gases, only small changes of resistance were observed directly after letting H_2_ into the chamber (Figure 11). This could be the effect of the change in oxygen concentration or RH after mixing H_2_ with the carrier gas. Hydrogen also has a very high heat conductivity, and at such high concentration can cause small local changes in the temperature of the ZnO resistive structure.

Due to the fact that the concentrations of hydrogen in the atmosphere of air were high, the saturation of the structure by hydrogen (H_2_, H^+^) is evident in the diagram in Figure 10b. The reaction of the ZnO nanostructure with 1% and 4% hydrogen is similar. At RT, the structure is not sensitive to the presence of hydrogen, even after optical excitation (Figure 11). The H_2_ molecules are not very reactive under standard conditions (the orbital s of the H_2_ molecule is complete).

#### 3.5.2. The Reaction of ZnO Nanostructures with Ammonia

**Figure 12:**
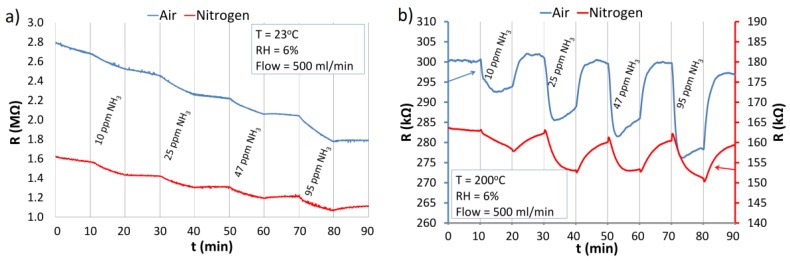
Reactions of the ZnO nanostructures with NH_3_ in air and nitrogen atmospheres at: (**a**) RT; (**b**) 200 °C.

The results of the investigations of the reaction of the structure to NH_3_ are shown in Figure 12. At RT, NH_3_ reacts with the ZnO nanostructures only slightly, decreasing the resistance of the ZnO structure (Figure 12a). In both atmospheres at RT, the reaction of ZnO nanostructures with ammonia is negligible and desorption is practically undetectable. At elevated temperature (200 °C), the ZnO nanostructures react distinctly with NH_3_ under both atmospheres (Figure 12b).

**Figure 13:**
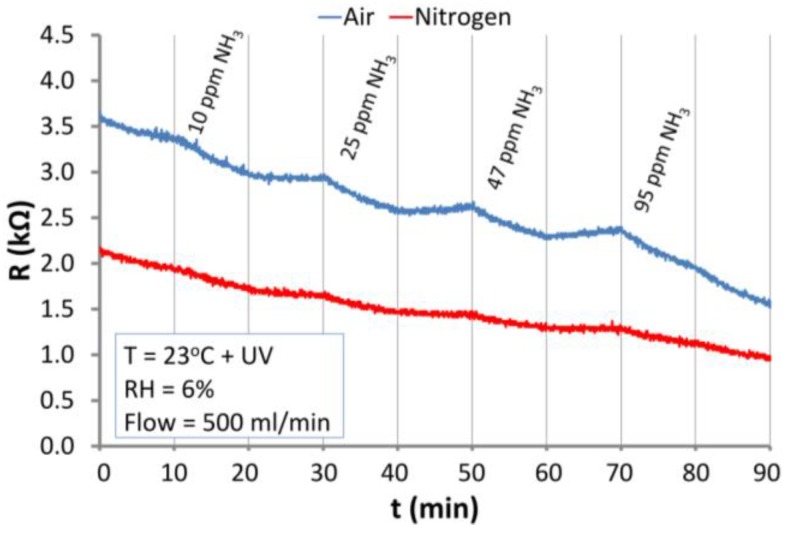
The response of the ZnO nanostructures to NH_3_ under continuous UV irradiation under atmospheres of air and nitrogen at RT (23 °C).

Similar to the case of H_2_, the interactions of ZnO nanostructures with NH_3_ were typical for reducing gases (resistance decreases). At the elevated temperature, a relatively fast desorption took place in the atmosphere of air and a comparably slow one in the nitrogen atmosphere. Figure 13 presents the sensitivity of the optically excited (UV) ZnO nanostructures exposed to ammonia in air and nitrogen atmospheres at RT.

It ought to be stressed that in spite of the similar character of the behavior of ZnO nanostructures exposed to NH_3_ and H_2_, the range of changes differs, taking into account the many times smaller concentrations of ammonia.

### 3.6. The Response of ZnO Nanostructures to Changes of the Humidity

**Figure 14:**
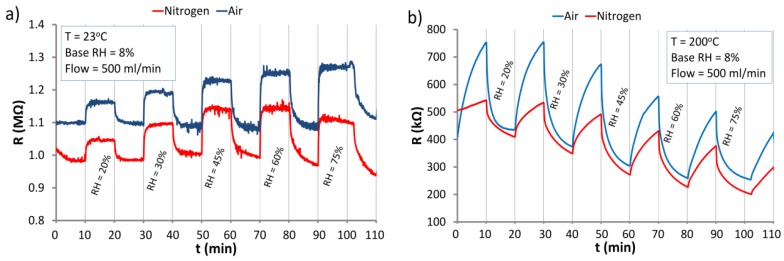
Reaction of the ZnO nanostructures to changes of the relative humidity (RH) level under atmospheres of nitrogen and air at: (**a**) RT; (**b**) 200 °C.

Figure 14 presents the reaction of ZnO nanostructures to changes of the RH level under atmospheres of air and nitrogen. The tests were performed at RT (23 °C) and elevated temperature of 200 °C.

The investigations (Figure 14) proved that the ZnO nanostructures are sensitive to humidity even at RT. It ought to be stressed that their response to RH reveals a different direction of resistance change, depending on the temperature at which the measurements were taken. At RT water is physically absorbed by ZnO nanostructures due the dipole character of the water molecules. The water molecules are absorbed by ZnO nanostructures as a result of van der Waals forces as well as, in some steps, by electrostatic forces. The polarity of H_2_O molecules changes the distribution of the electrical charges on the surface of ZnO nanostructures, therefore the resistance increases with increase in RH. This indicates that at low temperatures no dissociative adsorption of H_2_O molecules on ZnO surface takes place. The H_2_O dissociation can be observed at elevated temperatures from about 150 °C upwards [32]. This is connected with the supply of electrical charges to ZnO nanostructures and the neutralization of some part of the adsorbed oxygen molecules and lattice oxygen (caused by the reaction of H^+^ with O^−^). This leads to increasing the concentration of charge carriers. As an effect of this increase, the resistance of the structure drops, as can be seen in Figure 14b. At the temperature of about 200 °C, the effect of the dissociation of water on the electrical properties of ZnO nanostructures dominates. The investigations showed that at the temperature exceeding 200 °C humidity is chemisorbed (hydroxyl groups are joined to the ZnO nanostructures [31]).

**Figure 15:**
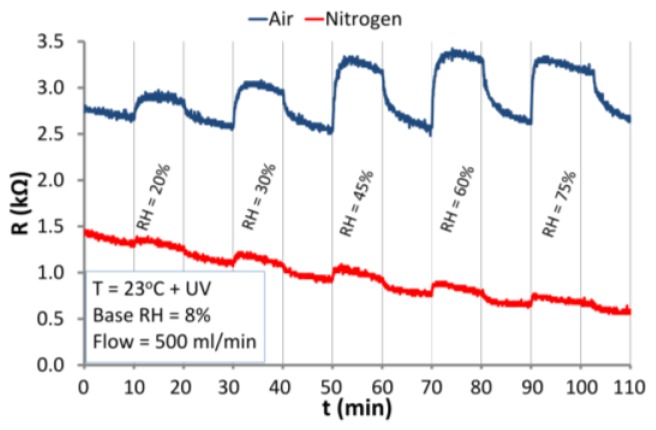
Responses of the ZnO nanostructures to RH changes under UV irradiation under atmospheres of air and nitrogen at RT (23 °C).

Under UV irradiation at RT (Figure 15), the sensor reacts similarly to the dark conditions at RT (Figure 15a). An increase in the RH level indicates the increase in the resistance of ZnO nanostructures. That proves that UV radiation does not cause dissociative chemisorption of water. As in the case of dark conditions under UV irradiation physical adsorption occurs. In the atmosphere of nitrogen, the structure reacts to RH changes weaker than in the atmosphere of air. This can be explained by the lower concentration of negatively charged oxygen adsorbed on the surface in the presence of nitrogen.

## 4. Summary and Conclusions

The aim of our study was to investigate the electrical properties of ZnO nanostructures obtained by the hydrothermal method composed of nanograins and nanowires (small and large grains) in different gaseous and external conditions. The effects of different gases, carrier gas (nitrogen and air), humidity, temperature, and electromagnetic irradiation on the resistance of the structure was examined. The investigated material showed promising NO_2_ sensing properties at RT with ultraviolet excitation and at elevated temperature (200 °C).

Our studies showed the role of atmospheric oxygen with respect to the sensitivity of the structure based on ZnO nanostructures on the effect of oxidizing and reducing gases. The experimental studies showed that the presence of oxygen in the gaseous atmosphere affects the electrical properties of the structure. The research showed that the sensitivity of ZnO nanostructures to gases depends on the temperature of these structures and their optical excitation (UV radiation).

**Figure 16:**
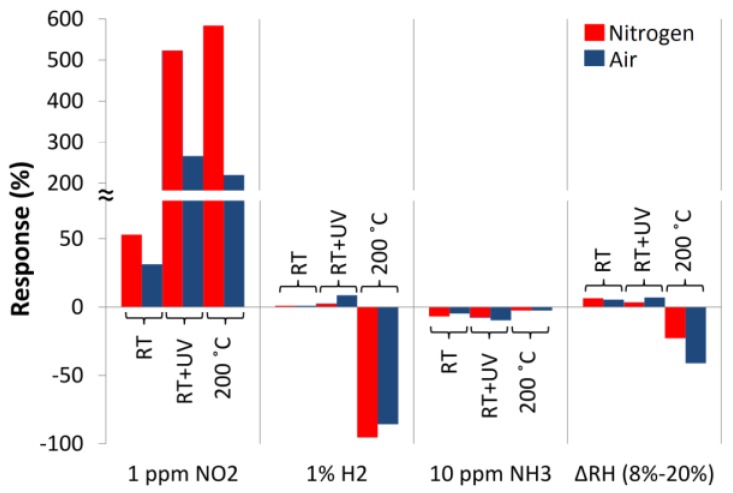
Comparison of the sensitivity of ZnO nanostructures to the action of selected gases.

The structure responses (Equation (1)) to all the investigated gases are summarized in Figure 16.

The results confirmed a strong increase in the responses of ZnO nanostructures at 200 °C. In dark conditions at RT the structure recovery was very weak or was not observed, whereas at 200 °C effective recovery occurred. The UV excitation of the ZnO nanostructures enabled effective recovery of the structure after its reaction with NO_2_ already at RT. Under UV condition at RT structure responses to NO_2_ are comparable with responses at 200 °C. The studies showed a limited impact of RH on the electrical resistance of ZnO nanostructures. At RT the impact of RH to the structure resistance is weaker than at elevated temperature. Also the different sorption mechanisms of the interaction of water molecules with ZnO nanostructures at RT and a temperature of 200 °C were observed (different directions of the reactions).

The interactions of ZnO nanostructures with gases showed typical reactions: their electrical conductivity increases when exposed to reducing gases (H_2_ and NH_3_), and decreases during exposure to oxidizing gases (O_2_ and NO_2_). Generally, at RT, the gas molecules interact with ZnO nanostructures primarily physically (except NO_2_), while at elevated temperatures, these interactions have the character of chemical adsorption [31]. It has to be stressed that the ZnO nanostructures are particularly sensitive to the presence of NO_2_ under both atmospheres (air and nitrogen). The response (Equation (1)) of ZnO nanostructure to the effect of NO_2_ at a temperature of 200 °C is more than 10^5^ times greater than the response of NH_3_, and more than 10^6^ times greater than that to H_2_ in the relation of 1 ppm. Thus the selectivity of the structure for NO_2_ is very good. What is more the selectivity to NO_2_ at RT with UV excitation increases in comparison to elevated temperature.

In Table 1 the comparison the response of the investigated structure to 1 ppm of NO_2_ to other ZnO nanostructure based gas sensors responses is presented. This comparison shows that the response of the presented structure is higher or comparable to other approaches. What is more under UV radiation high response is observed also at RT. This shows the possibility of detection of small concentrations of NO_2_ at RT with good sensitivity comparable to high temperature sensors (>200 °C). Operation of a potential sensor at RT ensures low energy consumption and improves the safety of its usage.

In comparison to the flower-like ZnO nanostructures which were investigated as a gas sensing material in our earlier work [21] the structure presented in this paper has better sensing properties (smaller noise level and much higher sensitivity to gases). Thanks to the use of a mixture of small grains and nanowires layers with improved adhesion and electrical stability were obtained. This proves that morphology has a very high impact on material sensing properties which are improved by the nanoparticle content in the investigated nanostructures.

It may be expected that the ZnO nanostructures allow the detection of low concentrations of NO_2_ in the range of ppb even at RT (with UV excitation). The investigated ZnO nanoparticles presents a high selectivity to NO_2_ and could be practically used as a sensor for this gas (e.g., for environmental monitoring). Such a high sensitivity of the ZnO nanostructures exposed to NO_2_ provides an opportunity to apply this type of ZnO nanostructures to detect vapors of explosives in the environment [54].

## Figures and Tables

**Table 1:** Comparison of responses of ZnO nanostructure based NO_2_ sensors with investigated ZnO nanostructures.

Material (Structure)	Synthesis Method	NO_2_ Concentration	Operating Temperature	Response (Carrier Gas)	Reference
ZnO (mixture of nanotubes and nanoparticles)	Hydrothermal	1 ppm	200 °C	600% (in nitrogen) 232% (in air)	Current paper
ZnO (mixture of nanotubes and nanoparticles)	Hydrothermal	1 ppm	RT (23 °C) + UV excitation	431% (in nitrogen) 341% (in air)	Current paper
ZnO (nanoporous thin film)	Sol-gel	100 ppm	200 °C	37% (in air)	[47]
ZnO (nanoflowers)	Hydrothermal	20 ppm	200 °C	9% (in air) 33% (in nitrogen)	[21]
ZnO + 2% TiO_2_ (nanocomposite)	Wet-chemical route	20 ppm	250 °C	58% (-)	[48]
ZnO (F doped thin film)	Chemical spraying method	1 ppm	200 °C	90% (in air)	[49]
ZnO (nanoparticles on sepiolite matix)	Participation onto sepiolite matrix	2.5 ppm	300 °C	1.1 (*R_a_*/*R_g_* in air)	[50]
ZnO (nanorods)	Plate method with hydrothermal deposition	10 ppm	200 °C	4 (*R_a_*/*R_g_* in air)	[51]
ZnO (nanoneedles)/ZnO (cacti-like structures)	Chemical route on glass substrate	200 ppm	200 °C	64% (in air)/89% (in air)	[52]
ZnO (Nanotetrapods)	Thermal evaporation controlled oxidation	20 ppm	300 °C	2000% (in air)	[53]

## References

[1] Li L., Zhai T., Bando Y., Golberg D. (2012). Recent progress of one-dimensional ZnO nanostructured solar cells. Nano Energy.

[2] Kayaci F., Vempati S., Donmez I., Biyikli N., Uyar T. (2014). Role of zinc interstitials and oxygen vacancies of ZnO in photocatalysis: A bottom-up approach to control defect density. Nanoscale.

[3] Cun T., Dong C., Huang Q. (2016). Ionothermal precipitation of highly dispersive ZnO nanoparticles with improved photocatalytic performance. Appl. Surf. Sci..

[4] Xu L., Zheng G., Wang J., Xian F., Liu Y. (2016). Stable co-emission of UV, green and red light in ZnO thin films with rapid annealing treatment. Opt. Int. J. Light Electron Opt..

[5] Saoud F.S., Plenet J.C., Henini M. (2015). Band gap and partial density of states for ZnO: Under high pressure. J. Alloys Compd..

[6] Ojha A.K., Srivastava M., Kumar S., Hassanein R., Singh J., Singh M.K., Materny A. (2014). Influence of crystal size on the electron–phonon coupling in ZnO nanocrystals investigated by Raman spectroscopy. Vib. Spectrosc..

[7] Morkoc H., Ozgur U. (2009). Zinc Oxide: Fundamentals, Materials and Device Technology.

[8] Chen M., Wang Z., Han D., Gu F., Guo G. (2011). High-sensitivity NO_2_ gas sensors based on flower-like and tube-like ZnO nanomaterials. Sens. Actuators B.

[9] Klingshirn C. (2007). ZnO: Material, physics and applications. ChemPhysChem.

[10] Yuan W., Liu A., Huang L., Li C., Shi G. (2013). High-performance NO_2_ sensors based on chemically modified graphene. Adv. Mater..

[11] Fanni L., Aebersold B.A., Alexander D.T.L., Ding L., Morales Masis M., Nicolay S., Ballif C. (2014). *c*-texture versus *a*-texture low pressure metalorganic chemical vapor deposition ZnO films: Lower resistivity despite smaller grain size. Thin Solid Films.

[12] Rai P., Khan R., Ahmad R., Hahn Y.-B., Lee I.-H., Yu Y.-T. (2013). Gas sensing properties of single crystalline ZnO nanowires grown by thermal evaporation technique. Curr. Appl. Phys..

[13] Tian Y., Li J., Xiong H., Dai J. (2012). Controlled synthesis of ZnO hollow microspheres via precursor-template method and its gas sensing property. Appl. Surf. Sci..

[14] Bai S., Sun C., Guo T., Luo R., Lin Y., Chen A., Sun L., Zhang J. (2013). Low temperature electrochemical deposition of nanoporous ZnO thin films as novel NO_2_ sensors. Electrochim. Acta.

[15] Thareja R.K., Saxena H., Narayanan V. (2005). Laser-ablated ZnO for thin films of ZnO and Mg*_x_*Zn_(1−*x*)_O. J. Appl. Phys..

[16] Kołodziejczak-Radzimska A., Jesionowski T., Krysztafkiewicz A. (2010). Obtaining zinc oxide from aqueous solutions of KOH and Zn(CH_3_COO)_2_. Physicochem. Probl. Miner. Process..

[17] Tong Y., Liu Y., Dong L., Zhao D., Zhang J., Lu Y., Shen D., Fan X. (2006). Growth of ZnO nanostructures with different morphologies by using hydrothermal technique. J. Phys. Chem. B.

[18] Neri G. (2015). First fifty years of chemoresistive gas sensors. Chemosensors.

[19] Pustelny T., Procek M., Maciak E., Stolarczyk A., Drewniak S., Urbańczyk M., Setkiewicz M., Gut K., Opilski Z. (2012). Gas sensors based on nanostructures of semiconductors ZnO and TiO_2_. Bull. Pol. Acad. Sci. Tech. Sci..

[20] Djurišić A.B., Chen X., Leung Y.H., Man Ching Ng A. (2012). ZnO nanostructures: Growth, properties and applications. J. Mater. Chem..

[21] Procek M., Pustelny T., Stolarczyk A., Maciak E. (2014). Studies of changes in electrical resistance of zinc oxide nanostructures under the influence of variable gaseous environments. Bull. Pol. Acad. Sci. Tech. Sci..

[22] Wang Y., Ma C., Sun X., Li H. (2002). Preparation of nanocrystalline metal oxide powders with the surfactant-mediated method. Inorg. Chem. Commun..

[23] Li P., Wei Y., Liu H., Wang X. (2005). Growth of well-defined ZnO microparticles with additives from aqueous solution. J. Solid State Chem..

[24] Djurišić A.B., Chen X.Y., Leung Y.H. (2012). Recent progress in hydrothermal synthesis of zinc oxide nanomaterials. Recent Pat. Nanotechnol..

[25] Zhao X., Li M., Lou X. (2014). Sol–gel assisted hydrothermal synthesis of ZnO microstructures: Morphology control and photocatalytic activity. Adv. Powder Technol..

[26] Liu X., Cheng S., Liu H., Hu S., Zhang D., Ning H. (2012). A survey on gas sensing technology. Sensors.

[27] Kumar R., Al-Dossary O., Kumar G., Umar A. (2015). Zinc oxide nanostructures for NO_2_ gas–sensor applications: A review. Nano Micro Lett..

[28] Fine G.F., Cavanagh L.M., Afonja A., Binions R. (2010). Metal oxide semi-conductor gas sensors in environmental monitoring. Sensors.

[29] Abdullah N.A., Khusaimi Z., Mohammad Rusop M. (2013). A review on zinc oxide nanostructures: Doping and gas sensing. Adv. Mater. Res..

[30] Procek M., Pustelny T. (2015). A study of gas sensing properties of ZnO nanostructures activated by UV light. Photonics Lett. Pol..

[31] Barsan N., Weimar U. (2001). Conduction model of metal oxide gas sensors. J. Electroceram..

[32] An W., Wu X., Zeng X.C. (2008). Adsorption of O_2_, H_2_, CO, NH_3_ and NO_2_ on ZnO nanotube: A density functional theory study. J. Phys. Chem. C.

[33] Struk P., Pustelny T., Gołaszewska K., Borysiewicz M.A., Piotrowska A. (2013). Gas sensors based on ZnO structures. Acta Phys. Pol. A.

[34] Gaman V.I. (2012). Physical principles of operation of oxidizing gas sensors based on metal oxide semiconductors. Russ. Phys. J..

[35] Ristić M., Musić S., Ivanda M., Popović S. (2005). Sol–gel synthesis and characterization of nanocrystalline ZnO powders. J. Alloys Compd..

[36] Chen X., Wong C.K.Y., Yuan C.A., Zhang G. (2013). Nanowire-based gas sensors. Sens. Actuators B.

[37] Yamazoe N., Shimanoe K. (2008). Theory of power laws for semiconductor gas sensors. Sens. Actuators B.

[38] Gao X., Li X., Yu W. (2005). Synthesis and characterization of flowerlike ZnO nanostructures via an ethylenediamine-meditated solution route. J. Solid State Chem..

[39] Pustelny T., Setkiewicz M., Drewniak S., MacIak E., Stolarczyk A., Urbańczyk M., Procek M., Gut K., Opilski Z., Pasternak I. (2013). The sensibility of resistance sensor structures with graphene to the action of selected gaseous media. Bull. Pol. Acad. Sci. Tech. Sci..

[40] Zhao J., Yang T., Liu Y., Wang Z., Li X., Sun Y., Du Y., Li Y., Lu G. (2014). Enhancement of NO_2_ gas sensing response based on ordered mesoporous Fe-doped In_2_O_3_. Sens. Actuators B.

[41] Mishra S., Ghanshyam C., Ram N., Bajpai R.P., Bedi R.K. (2004). Detection mechanism of metal oxide gas sensor under UV radiation. Sens. Actuators B.

[42] Yamazoe N., Suematsu K., Shimanoe K. (2012). Extension of receptor function theory to include two types of adsorbed oxygen for oxide semiconductor gas sensors. Sens. Actuators B.

[43] Procek M., Pustelny T. (2013). Analysis of the responses of metal-Oxide semiconductor nanostructures to nitrogen dioxide. Acta Phys. Pol. A.

[44] Mikhaylov R.V., Lisachenko A.A., Shelimov B.N., Kazansky V.B., Martra G., Coluccia S. (2013). FTIR and TPD study of the room temperature interaction of a NO–Oxygen mixture and of NO_2_ with titanium dioxide. J. Phys. Chem. C.

[45] Chen H., Liu Y., Xie C., Wu J., Zeng D., Liao Y. (2012). A comparative study on UV light activated porous TiO_2_ and ZnO film sensors for gas sensing at room temperature. Ceram. Int..

[46] Meyer B. (2003). First-principles study of the polar O-terminated ZnO surface in thermodynamic equilibrium with oxygen and hydrogen. Phys. Rev. B.

[47] Chougule M.A., Sen S., Patil V.B. (2012). Fabrication of nanostructured ZnO thin film sensor for NO_2_ monitoring. Ceram. Int..

[48] Vyas R., Sharma S., Gupta P., Vijay Y.K., Prasad A.K., Tyagi A.K., Sachdev K., Sharma S.K. (2013). Enhanced NO_2_ sensing using ZnO-TiO_2_ nanocomposite thin films. J. Alloys Compd..

[49] Şennik E., Kerli S., Alver Ü., Öztürk Z.Z. (2015). Effect of fluorine doping on the NO_2_-sensing properties of ZnO thin films. Sens. Actuators B.

[50] Hassan M., Afify A.S., Tulliani J.M. (2016). Synthesis of ZnO nanoparticles onto sepiolite needles and determination of their sensitivity toward humidity, NO_2_ and H_2_. J. Mater. Sci. Technol..

[51] Harale N.S., Kamble A.S., Tarwal N.L., Mulla I.S., Rao V.K., Kim J.H., Patil P.S. (2016). Hydrothermally grown ZnO nanorods arrays for selective NO_2_ gas sensing: Effect of anion generating agents. Ceram. Int..

[52] Pawar R.C., Lee J.W., Patil V.B., Lee C.S. (2013). Synthesis of multi-dimensional ZnO nanostructures in aqueous medium for the application of gas sensor. Sens. Actuators B.

[53] Calestani D., Zha M., Mosca R., Zappettini A., Carotta M.C., Di Natale V., Zanotti L. (2010). Growth of ZnO tetrapods for nanostructure-based gas sensors. Sens. Actuators B.

[54] Procek M., Stolarczyk A., Pustelny T., Maciak E. (2015). A study of a QCM sensor based on TiO_2_ nanostructures for the detection of NO_2_ and explosives vapours in air. Sensors.

